# Ti_2_C-TiO_2_ MXene Nanocomposite-Based High-Efficiency Non-Enzymatic Glucose Sensing Platform for Diabetes Monitoring

**DOI:** 10.3390/s22155589

**Published:** 2022-07-26

**Authors:** Vinod Kumar, Sudheesh K. Shukla, Meenakshi Choudhary, Jalaj Gupta, Priyanka Chaudhary, Saurabh Srivastava, Mukesh Kumar, Manoj Kumar, Devojit Kumar Sarma, Bal Chandra Yadav, Vinod Verma

**Affiliations:** 1 Stem Cell Research Centre, Department of Hematology, Sanjay Gandhi Post Graduate Institute of Medical Sciences, Lucknow 226014, UP, India; jgupta@sgpgi.ac.in; 2 Department of Biomedical Engineering, Shobhit Institute of Engineering & Technology (Deemed To-Be-University), Meerut 250110, UP, India; sudheesh.shukla@shobhituniversity.ac.in; 3 Department of Solar Energy and Environmental Physics, The Swiss Institute of Dryland, Environmental and Energy Research, The Jacob Blaustein Institutes for Desert Research, Ben-Gurion University of the Negev, Midreshet Ben-Gurion 8499000, Israel; kmmeenakshi6@gmail.com; 4 School of Physical & Decision Sciences, Babasaheb Bhimrao Ambedkar University (A Central University), Lucknow 226025, UP, India; chaudharypriyanka702@gmail.com (P.C.); balchandra_yadav@rediffmail.com (B.C.Y.); 5 Department of Applied Sciences & Humanities, Rajkiya Engineering College Ambedkar Nagar, Dr. A.P.J. Abdul Kalam Technical University, Ambedkar Nagar 224122, UP, India; saurabhnpl@gmail.com; 6 Department of Zoology, Babasaheb Bhimrao Ambedkar University (A Central University), Lucknow 226025, UP, India; mukeshcomm@gmail.com; 7 Indian Council of Medical Research—National Institute for Research in Environmental Health, Bhopal Bypass Road, Bhouri, Bhopal 462030, MP, India; manoj15ndri@gmail.com (M.K.); devojit.sarma@icmr.gov.in (D.K.S.)

**Keywords:** diabetes, Ti_2_C-TiO_2_ MXene, nanocomposite, non-enzymatic glucose sensor (NEGS)

## Abstract

Diabetes is a major health challenge, and it is linked to a number of serious health issues, including cardiovascular disease (heart attack and stroke), diabetic nephropathy (kidney damage or failure), and birth defects. The detection of glucose has a direct and significant clinical importance in the management of diabetes. Herein, we demonstrate the application of *in-situ* synthesized Ti_2_C-TiO_2_ MXene nanocomposite for high throughput non-enzymatic electrochemical sensing of glucose. The nanocomposite was synthesized by controlled oxidation of Ti_2_C-MXene nanosheets using H_2_O_2_ at room temperature_._ The oxidation results in the opening up of Ti_2_C-MXene nanosheets and the formation of TiO_2_ nanocrystals on their surfaces as revealed in microscopic and spectroscopic analysis. Nanocomposite exhibited considerably high electrochemical response than parent Ti_2_C MXene, and hence utilized as a novel electrode material for enzyme-free sensitive and specific detection of glucose. Developed nanocomposite-based non-enzymatic glucose sensor (NEGS) displays a wide linearity range (0.1 µM-200 µM, R^2^ = 0.992), high sensitivity of 75.32 μA mM^−1^ cm^−2^, a low limit of detection (0.12 μM) and a rapid response time (~3s). NEGS has further shown a high level of repeatability and selectivity for glucose in serum spiked samples. The unveiled excellent sensing performance of NEGS is credited to synergistically improved electrochemical response of Ti_2_C MXene and TiO_2_ nanoparticles. All of these attributes highlight the potential of MXene nanocomposite as a next-generation NEGS for on the spot mass screening of diabetic patients.

## 1. Introduction

Diabetes is one of the fastest rising worldwide health emergencies in the 21st century [1]. Being a metabolic disorder, diabetes is characterized by abnormal blood glucose levels compared with the therapeutic range of 80–120 mg dL^−1^ (4.4–6.6 mM) [2]. The disorder has accounted for around 537 million people in 2021, and the figure is expected to rise to 643 million by 2030 [1]; moreover, it is linked to a number of serious health issues, including cardiovascular disease (heart attack and stroke), diabetic nephropathy (kidney damage or failure), and birth defects [3]. In order to manage the disorder properly, it is essential to develop highly efficient, robust, and cost-effective diagnostic platforms to monitor glucose levels on a regular basis [4]. Over the last couple of decades, various types of sensing modalities including optical and electrochemical methods were developed for precise glucose detection [4,5]. Among these, the electrochemical sensors have received wide popularity [6], and are categorized as enzymatic and non-enzymatic sensors [5,6]. In spite of their widespread acceptance [7], the performance of enzymatic glucose sensors is significantly hampered by many environmental factors such as pH, temperature, hazardous compounds, and humidity, which have negatively impacted their cost and breadth of applications [8]. On the other hand, non-enzymatic glucose sensors (NEGS), have made substantial progress owing to their direct electro-oxidation capabilities, ease to prepare, better endurance, a longer lifetime, and notable selectivity, sensitivity, and affordability [7].

In recent years, various engineered materials (such as metals and their compounds, inorganic carbon nanomaterials, conductive polymers, two-dimensional sulphides and metal–organic frameworks) have been utilized as electrode materials, either separately, or in combinations to improve the sensing performances of platforms/devices [7]. Nanocomposites of carbon and transition metal dichalocogenides, in particular, have demonstrated enormous possibilities for the development of efficient glucose non-enzyme sensors [7]; however, the time-consuming synthesis approaches of these nanocomposites and unexpectedly low sensing performance have generated an interest in exploring new material combinations for NEGS [9]. Consequently, MXene and its nanocomposite have emerged as viable alternatives for a variety of analytical purposes, including NEGS [9,10]. MXenes, in general, are novel and a new class of two-dimensional inorganic nanomaterials with a few atomic layer thickness of transition metal carbides, nitrides, or carbonitrides such as titanium carbide (Ti_3_C_2_) and titanium carbonitride (Ti_2_CN) [9]. MXene’s unique structure has endowed them with several exceptional optical and electrochemical properties, making them suitable for wide range of applications [11]. Furthermore, MXene’s biocompatibility permits them to be used in a variety of biological activities [10]. The recently reported high NEGS performance of a Ti_3_C_2_ MXene nanocomposite with NiCo-LDH [12] has paved the way for more study using MXenes nanocomposite towards NEGS [12].

In the current study, we have explored the electrochemical performance of *in-situ* synthesized MXene nanocomposite i.e., Ti_2_C-TiO_2_, in developing highly efficient NEGS. The developed NEGS has shown quite an impressive sensing performance for glucose even in bio-fluids, which is credited to the high surface area of nanocomposite that allows the rapid diffusion of ions for easy access to glucose to proceed with the electrochemical reaction at a faster rate. In addition, the hydrophobicity of MXene offers nanocomposite to make full contact with the electrolyte for an adequate redox reaction.

## 2. Experimental Details

### 2.1. Materials and Reagents

Ti_2_AlC powders (bulk) were purchased from Nanochemzone, Canada. Lithium Fluoride (LiF), Hydrogen Fluoride (HF), Hydrogen Chloride (HCl), Hydrogen Peroxide (H_2_O_2_), Sodium Hudroxide (NaOH), D-Glucose, Ascorbic Acid, Uric Acid, Dopamine, Serum samples other solvents and buffers were procured from Sigma Aldrich, India.

### 2.2. Synthesis and Characterization of Ti_2_C MXene and Ti_2_C-TiO_2_ MXene Nanocomposite

**Figure 1:**
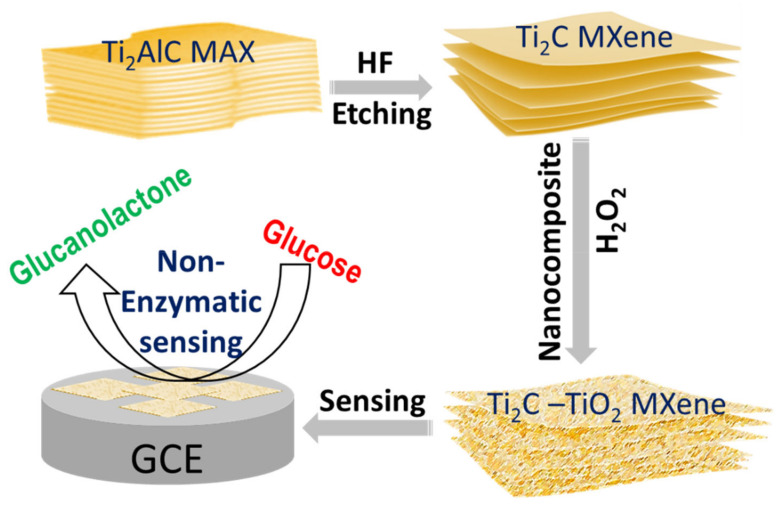
Scheme showing synthesis of Ti_2_C-TiO_2_ MXene nanocomposite and its application in non-enzymatic glucose sensing (NEGS).

Ti_2_C MXene nanosheets were prepared via selective etching of the Al layer from MAX phase of Ti2AlC following a protocol reported in a previous study [13]. Ti2AlC powder was reacted with 10% HF for 10 h at room temperature. The resulting black-colored suspension of Ti_2_C MXene nanosheets was washed 6 times with deionized water (DI) by centrifugation of the samples at 5000 rpm for 5 min each time till the pH changes from acidic to neutral (pH-7.0); finally the sample was filtered and dried at 60 °C for 24 h under vacuum to obtain Ti_2_C MXene nanosheets. For the preparation of Ti_2_C-TiO_2_ MXene nanocomposite, we have used a protocol report earlier by Ahmed et al., with slight modifications [14]. In a typical reaction, 0.5 g of Ti2C MXene nanopowder was refluxed with 50 mL of water using a magnetic stirrer (for 10 min), followed by a reaction with of 5 mL of different weight % (5, 10, 15 wt.%) of H_2_O_2_ for 10, 15, 20 min for each in separate sets of reactions. The resultant products were washed several times with DI water (with the help of centrifugation) and dried under vacuum for 24 h before further experiments. Figure 1 is the schematic representation of synthesis and application of MXene nanocomposite as NEGS. The powder of the prepared Ti_2_C MXene and its nanocomposite were characterized by using a powder X-ray diffractometer (XRD, Bruker, D8 ADVANCE) with Cu Kα radiation (λ = 0.15406 nm), Raman spectroscopy using a LabRam Aramis Raman spectrometer with a diode-pumped solid-state blue laser having an excitation wavelength of 473 nm and Fourier Transform Infrared Spectroscopy (FTIR) with reflectance transmission (Perkin Elmer). The surface features and microstructure of Ti_2_C MXene and its nanocomposite were examined under scanning electron microscope (SEM, FEI). For SEM, and Raman analysis, samples were drop cast on a silicon wafer, while for FTIR KBr pellet saturated with materials was used.

### 2.3. Fabrication and Characterization of MXene Nanocomposite Electrode and Its Application for NEGS

Prior to experiments, the glassy carbon electrode (GCE) was polished to a mirror-like surface using 1 µm and 0.05 µm dimeter of alumina powder followed by ultrasonic cleaning in ultrapure water and ethanol [15]. Furthermore, 1 mg mL^−1^ concentration of each Ti_2_C-TiO_2_ MXene nanocomposites was prepared by dispersing them separately in a solution of ethanol, and ultrapure water (1:1: V:V) containing 20 µL 0.5wt% Nafion. Thereafter, 5 µL (optimized concentration, Supplementary Figure S2) of each solution was placed on the mirror-like surface of the GCE and allowed to dry at room temperature. Cyclic voltammetry (CV), was performed to compare the electrochemical properties of the nanocomposites modified electrodes in 0.1 M NaOH using Ag/AgCl as reference and Pt as a counter electrode. In CV characterization, a nanocomposite synthesized using 10% H_2_O_2_ with a reaction time of 15 min, has shown a comparatively higher electrochemical response than others, (Supplementary Figure S2). Therefore, the same nanocomposite was tested for enzyme-free sensing of glucose using the differential pulse voltammetry technique (DPV), and chronoampertometry was performed at 0.1 M NaOH. The sensing performances were studied in an alkaline medium because, in optimization of electrochemical response, the MXene nanocomposite has shown comparatively high signals (0.30 times) in an alkaline medium than in a neutral solution. Institutional ethical approval was waived off by a duly constituted committee of Shobhit Institute of Engineering & Technology, India, prior to working with commercially available human serum samples.

## 3. Results and Discussion

### 3.1. Characterization of Ti_2_C MXene and Ti_2_C-TiO_2_ MXene Nanocomposite

#### 3.1.1. X-ray Diffraction (XRD) Analysis

**Figure 2:**
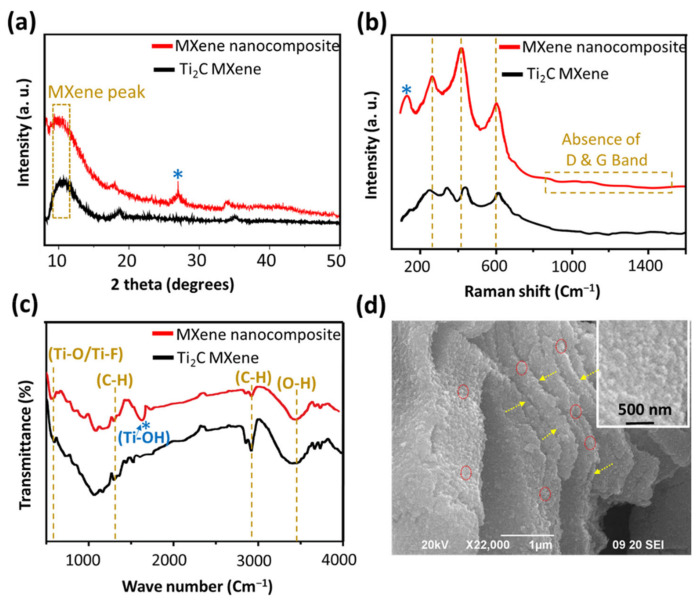
Characterization of Ti_2_C MXene and Ti_2_C-TiO_2_ MXene nanocomposite (synthesized by controlled oxidation of MXene nanosheets using 10% H_2_O_2_ for 15 min of reaction), (**a**) XRD patterns, (**b**) Raman spectra, (**c**) FTIR and (**d**) SEM image of a typical nanocomposite (inset showing *in-situ* synthesized TiO_2_ nanoparticles from Ti_2_C MXene), arrows show the sheets while circles indicate the position of TiO_2_ nanoparticles.

The XRD patterns of as-prepared Ti_2_C MXene and Ti_2_C-TiO_2_ MXene nanocomposite (reaction condition: 10% H_2_O_2_ with 15 min) powders are shown in Figure 2a. The (0002) peak of the Ti_2_AlC MAX phase shifts towards lower angles in the Ti_2_C MXene, indicating the elimination of Al layer and a rise in the *c* lattice parameter [16]. Furthermore, the (0002) peak, also known as the MXene peak, broadens in comparison to the sharper MAX phase peak, which corresponds to decreased structural order [16]. The XRD spectrum of nanocomposite shows the presence of the anatase phase of TiO_2_. The (101) plane of anatase TiO_2_ (JCPDS number 00-021-1272) [17] corresponds to a strong peak at 2*θ*~25°. Despite the presence of anatase, the (0002) peak of the MXene phase is also evident, indicating that the resultant product is primarily the MXene phase.

#### 3.1.2. Raman Analysis

Figure 2b shows the Raman spectra of Ti_2_C MXene and its nanocomposite. The nanocomposite exhibited a major peak of about 150 cm^−1^, which corresponds to anatase phase of TiO_2_ [18]. The other three vibrational modes at 250, 400, and 600 cm^−1^ are assigned to nonstoichiometric titanium carbide [19]. Furthermore, the Raman band centered at 400 cm^−1^ shifted towards higher wavelengths after H_2_O_2_ treatment, which can be attributed to layer emaciation [17]. As previously stated, a decrease in layer thickness causes the band position to shift to higher energy due to modest stiffening of the bonds [17]. Another intriguing finding in Raman spectra of nanocomposite is the disappearance of D and G bands. In a previous study, it was reported that flash oxidation of MXene resulted into the formation of nanocrystalline TiO_2_ on disordered carbon sheets [18]; however, the lack of D and G bands in our samples implies that our end product is nanocrystalline TiO_2_ grown over MXene (Ti_2_CTx) sheets [18].

#### 3.1.3. Fourier Transform Infrared Spectroscopy (FTIR) Analysis

Fourier Transform Infrared Spectroscopy (FTIR) was used to investigate the generation of various functional groups during the process of etching and formation of nanocomposite. The bands corresponding to functional groups -OH, -O-, and F- are common in FTIR spectra of both MXene and its nanocomposite, while in nanocomposite specific band at 1600 cm^−1^ is due TiO_2_ highlighting a successful synthesis of nanocomposite.

#### 3.1.4. Surface Morphological Analysis

Figure 2d is a SEM image of a nanocomposite showing the opening of layers and anchorage of TiO_2_ nanocrystals over and under the layers. Synthesized MXene nanocomposites were found to be multilayered (2.5 µM) with lateral dimensions of ~1 µM. A uniform distribution of TiO2 nanocrystals with size of ~100 nm were found throughout the surface and at the edges of Ti2C MXene (Supplementary Figure S1) Moreover, SEM elemental analysis also confirms the composition of nanocomposite (Supplementary Figure S1); this finding is supported by the earlier stated XRD, Raman, and FTIR studies.

The oxidation reaction of Ti_2_C MXene by H_2_O_2_ can be written as follows [14]:

*a*Ti_2_CT_x_ + H_2_O_2_ → *b*TiO_2_ + (1 − *b*)Ti_2_CT_x_ + Coy (1)

## 4. Application as Non-Enzymatic Glucose Sensor (NEGS)

### 4.1. Electrochemical Properties of Ti_2_C MXene and T_i2_C-TiO_2_ MXene Nanocomposite

**Figure 3:**
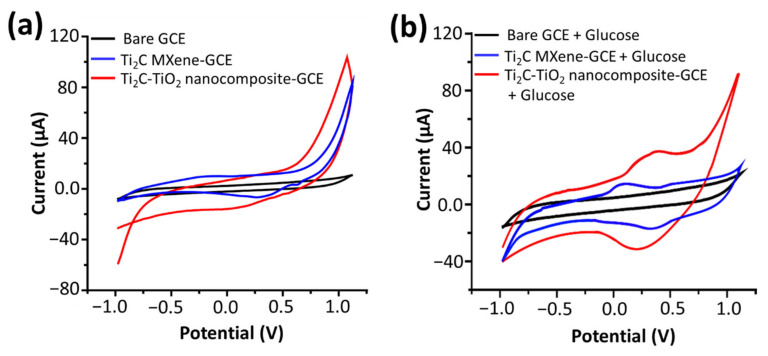
Cyclic voltammogram (CV) of bare, and modified GCE (with Ti_2_C MXene and Ti_2_C-TiO_2_ MXene nanocomposite), (**a**) without, and (**b**) with 0.1 mM glucose, recorded in 0.1 M NaOH (pH 13) at 100 mV s^−1^.

Electrochemical measurements were carried out in 0.1 M NaOH using an Autolab potentiostat/galvanostat (Eco Chemie, The Netherlands) using a three-electrode setup. CV of the bare GCE (black curve), Ti_2_C MXene (blue curve) and Ti_2_C-TiO_2_ MXene nanocomposite modified electrodes (red curve) at a sweeping potential of −1 to +1 Volts (scan rate of 100 mV s^−1^) are shown in Figure 3a. MXene nanocomposite modified electrode exhibited a cathodic current of (20 µA), while it was measured to be (10 µA) with MXene only, and almost negligible response with bare GCE. Thus, a clear two-fold increase in cathodic current in nanocomposite was recorded than that of MXene. The improved electrochemical activity of nanocomposite is due the synergism between MXene and TiO_2_ nanoparticles. To study the electrochemical mechanism, the CV response of MXene nanocomposites were recorded under various scan rates (50 mV s^−1^–500 mV s^−1^ in 0.1 M NaOH) and the results are displayed in Figure S3a (Supplementary Materials). As shown in Figure S3b, cathodic peak currents (I_c_) have a linear relationship with the square root of the scan rates, showing that an electrochemical reaction process driven by diffusion occurred on the modified electrodes. With the addition of 0.1 mM glucose, the anodic and cathodic peak current of Mxenes and nanocomposite electrodes increased significantly, which can be ascribed to the oxidation of glucose (Figure 3b); moreover, the anodic peak current of nanocomposite (38 µA) is ~2 times larger than that of MXene (20 µA), which implies that nanocomposite owns better catalytic performance compared with MXene. Excellent electrochemical activity, as well as the high surface area of the nanocomposite, accelerates transfer rate of electrons and enhances the catalytic oxidation of glucose thus improving the performance NEGS. The anticipated mechanism of the glucose oxidation on the surface of nanocomposite (in alkaline solution) began with the deprotonation of glucose. The current response of the nanocomposite was measured to be significantly higher than that of Ti_2_C MXene with 0.1 mM glucose. Still, we are trying to explore the glucose oxidation mechanism by nanocomposite; however, the following chemical formulas show the glucose oxidation mechanism (reaction shown in Equations (2)–(6)) for titanium dioxide [20].

2TiO_2_ + C_6_H_12_O_6_ (glucose) → 2TiOOH + C_6_H_10_O_6_ (gluconolactone) (2)

2Ti(IV) + C_6_H_12_O_6_ (glucose) → 2Ti(II) + C_6_H_10_O_6_ (gluconolactone) + 2H_2_O_2_ (3)

C_6_H_10_O_6_ (gluconolactone) + H_2_O → 2H^+^ + C_6_H_12_O_7_ (gluconate) (4)

2Ti(II) → 2Ti(IV) + 2e-(5)

The electrons transported during the electrochemical catalytic oxidation process are calculated using Brown–Anston model (Equation (6))

I_p_ = n^2^F^2^I*Av/4RT (6)

where, n is the number of electrons transferred, F is Faraday constant (96,485.5 C mol^−1^), I* is the surface concentration (mol cm^−2^), A is the surface area of the electrode (0.5 cm^2^), V is the scan rate (100 mVs^−1^), R is gas constant (8.134 mol^−1^ K) and T is the room temperature (298 K). Therefore, n was calculated to be 2.0; thus, the whole electrocatalytic process of glucose involved two electrons participating in the procedure.

### 4.2. Non-Enzymatic Glucose Sensing

**Figure 4:**
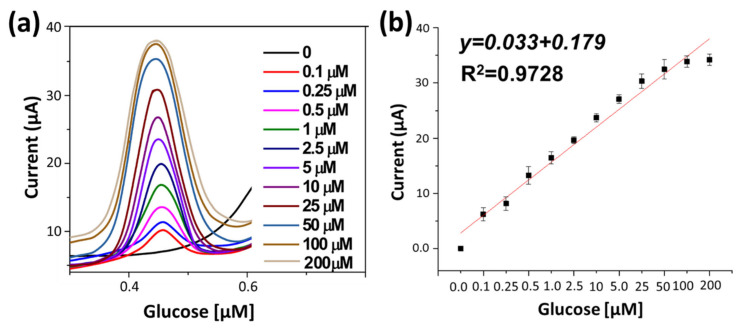
(**a**) Differential pulse voltammogram (DPV) of developed NEGS with different concentration glucose in 0.1 M NaOH (pH 13), (**b**) Calibration curve (current vs. glucose concentration).

Performance of developed NEGS was further examined by differential pulse voltammetry technique (DPV), because it is a sensitive technique and less likely to be impacted by capacitive current than CV. DPV only measures the difference in current between the beginning and end of each pulse. The faradaic current response NEGS with a range of glucose concentrations (0.1–200 µM) were recorded. Figure 4a, represent the corresponding current responses when measured in an alkaline medium (0.1M NaOH solution) with the potential range of 0–0.7 V under the optimization conditions, the anodic peak currents appeared at approximately +0.45 V versus the Ag/AgCl reference electrode. As shown in Figure 4b, the typical peak current–concentration-response curve followed a linear relationship.

The sensitivity of NEGS was measured to be 75.32 μA mM^−1^ cm^−2^ with limit of detection (LOD) 0.12 μM (S/N = 3).

**Figure 5:**
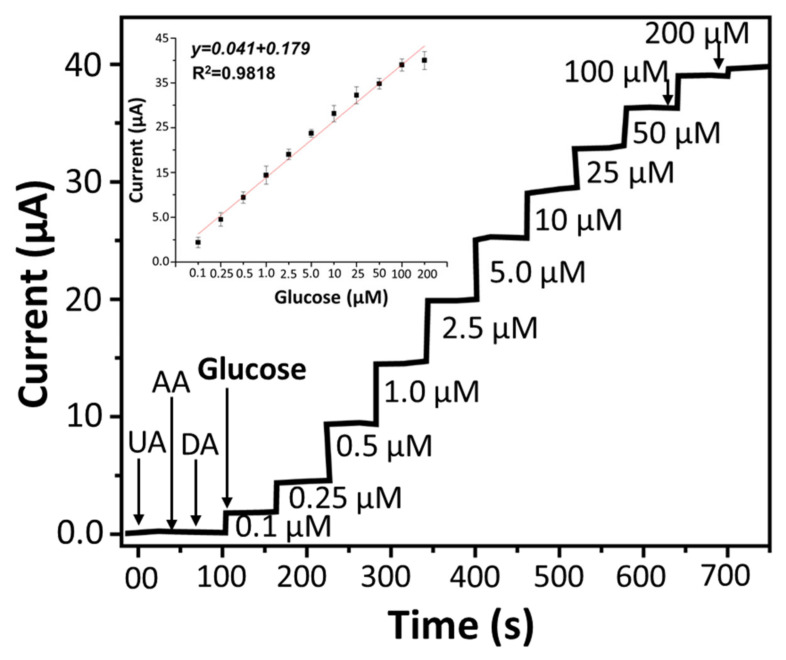
Amperometric response of NEGS with different conc. of glucose added in 0.1 M NaOH (pH 13) with 30 s of intervals. NEGS show no current response upon addition of different interference such as uric acid (UA), ascorbic acid (AA), Dopamine (DA) at a concentration of 50 μM of each, while a linear current response with varying glucose concentrations were clearly observed (inset calibration graph of current response vs. glucose concentration).

In order to re-confirm the efficiency of NEGS chronoamperometry measurements were carried out with varying concentrations (0.1–200 µM) of glucose (added in 0.1 M NaOH solution every 30 s) at a voltage of 0.45 V (Optimized potential, Supplementary Figure S4). The current increases as glucose is added to the alkaline solution, and it only takes 3 s for the current to reach a steady state (Figure 5). The calibration curve clearly shows a linear increase in current with glucose concentrations (Figure 5 inset).

The sensitivity of NEGS was calculated to be 73.79 μA mM^−1^ cm^−2^ and the LOD is 0.14 μM (S/N = 3). When compared to earlier works, developed NEGS has exhibited higher analytical performance (Table 1). The exhibited high sensing performances of developed NEGS can be attributed to the composition and structure of the nanocomposite in general, and specifically: (i) The high surface area of nanocomposite offers an for easy electrolyte diffusion and full contact with active materials; (ii) The combination of nanosheets (Ti_2_C MXene) and nanoparticles (TiO_2_) significantly improves the electrochemical activity of the nanocomposite and accelerates electron transfer rate.

### 4.3. Specificity, Reproducibility and Stability of the Sensor

**Figure 6:**
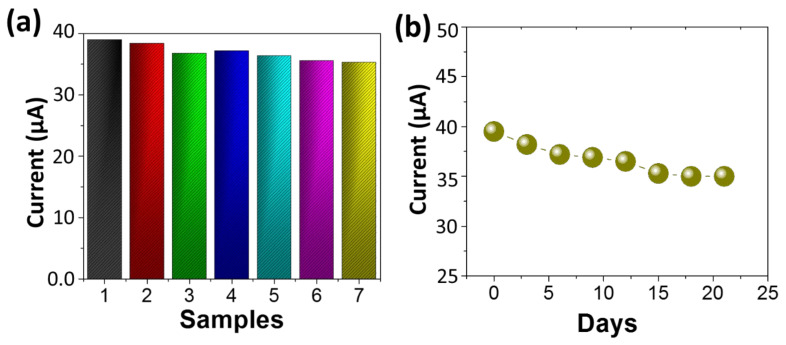
(**a**) Reproducibility of the fabricated 07 NEGS under identical condition, exposed with 0.1 mM glucose, (**b**) Stability of NEGS over period of 21 days.

For a sensor specificity or selectivity is an important performance parameters. The selectivity of developed NEGS was determined in presence of ascorbic acid (AA), uric acid (UA), and dopamine (DA) which co-exist (with glucose) and substantially interfere with glucose determination. Amperometric response of NEGS after the addition of 0.1 µM glucose and 50 µM interfering species in 0.1 M NaOH was measured. The current response of the NEGS towards AA, UA, and DA were found to be substantially lower than that of Glu (Figure 5), highlighting the high selectivity of NEGS towards glucose. The reproducibility of NEGS was further determined by monitoring the current response of 07 electrode (manufactured under identical conditions) after exposure of 0.1 mM glucose and relative standard deviation (RSD) was measured to be 4%, (Figure 6a), indicating that the NEGS has excellent repeatability. Furthermore, the stability of NEGS was investigated by dissolving 0.1 mM glucose in 0.1 M NaOH solution. As shown in Figure 6b, the current response remained 95.1% of the initial value after 07 days and 93.5% of the initial value after 15 days, suggesting that NEGS has reasonably good durability as an enzyme-free glucose sensor.

### 4.4. Real Sample Analysis

The accuracy of developed NEGS was compared with a commercially available glucometer in human serum samples (available commercially). Initially, the basal glucose level in serum was set to be 0.15 ± 0.01 mM with the help of commercially available glucometer. The same serum samples was used for the analysis with NEGS (through DPV measurements) and, the average glucose concentration as was found to be 0.14mM+/− 0.03 mM, with an RSD of 2.91 percent (Table 2). The accuracy of the NEGS was further tested with the serum samples (used above) spiked with 1 mM, 2 mM, 3 mM, 4 mM and 5 mM of glucose. NEGS (DPV measurements) analytical results are shown in Table 2. The results have shown a close matching to the results obtained through a glucometer. Thus, NEGS possesses a high level of detection accuracy for glucose in bio-fluids also. A 10% loss in sensitivity was noticed with the neutral solution and the detection limit still fulfills the requirement for clinical applications.

## 5. Conclusions

We successfully demonstrated the fabrication of Ti_2_C-TiO_2_ MXene nanocomposite based an efficient non-enzymatic glucose sensors (NEGS). The nanocomposite was synthesized in facile manner at room temperature, and thoroughly characterized by XRD, Raman, FTIR and SEM. When compared to earlier studies, the developed NEGS outperforms them in terms of wide linear range (0.1–200 µM), low LOD (0.12 µM), fast response (3 s), excellent selectivity, reproducibility, stability, along with notable recovery of glucose from spiked serum samples. The improved sensing performances of developed NEGS is attributed to the mutual contribution of MXene and TiO_2_ nanoparticles. The excellent electrochemical performance of partially oxidized MXene makes it a promising candidate for building novel point-of-care electrochemical devices for NEGS in clinical samples.

## Figures and Tables

**Table 1:** Comparison of the developed sensor with previous studies.

Developed Glucose Sensor	Sensitivity [μA cm^−2^ mM^−1^]	Linear Range [mM]	Reference
GOx/n-TiO_2_/PANI/GCE	6.31	0.02–6.0	[21]
Pt/CNTs/TiO_2_ NTAs	0.24	0.006–1.5	[22]
GOx/TiO_2_/CNTs	11.3 ± 1.3	Up to 3.0	[23]
TiO_2_-SWCNT NWS	5.32	0.010–1.42	[24]
Cu_2_O/TiO_2_	14.56	3.0–9.0	[25]
GOx/Ag/TiO_2_ NTAs	0.39	0.1–4.0	[26]
GOx/Pt/Gr/TiO_2_ NTAs	0.94	0.1–8.0	[27]
AuNPs-TiO_2_ NT	-	0.40–8.0	[28]
TiO_2_-GR	6.20	0–8.0	[29]
GOD/1DH S-TiO_2_	9.9	0.2–1.0	[30]
GOD/HNF-TiO_2_/GC	32.6	0.002–3.17	[31]
GCE/TiO_2_ NW/PAPBA-Au TNC	66.8	0.5–11.0	[32]
MXene/NiCo-LDH	64.75	0.002–4.096	[12]
**NEGS (Ti_2_C-TiO_2_ MXene nanocomposite)**	**75.32**	**0.0001–0.2**	**This work**

**Table 2:** Comparison of sensing performance of developed NEGS with earlier reported non-enzymatic sensors (Each experiment was performed in triplicate, and data shown below are the average of them).

Sample	Spiked Glucose [mM]	Concentrations (mM)		% Recovery	% RSD
		Detected by Glucometer	Detected by Developed NEGS		
Human serum samples	0	0.15 ± 0.01	0.14 ± 0.03	99.8	
	1	1.15 ± 0.05	1.1412 ± 0.02	99.94	
	2	2.15 ± 0.02	2.1475 ± 0.04	99.96	
	3	3.149 ± 0.3	3.148 ± 0.02	99.98	2.91
	4	4.148 ± 0.01	4.151 ± 0.02	100.23	
	5	5.147 ± 0.07	5.1481 ± 0.03	100.11	

## Data Availability

Not applicable.

## Supplementary Materials

The following supporting information can be downloaded at: https://www.mdpi.com/article/10.3390/s22155589/s1, Figure S1: SEM of synthesized MXene nanocomposite and elemental analysis; Figure S2: (a) Electrochemical characterization of synthesized nanocomposites, (b) Optimization of electrochemical response ofMXene nanocomposite; Figure S3: (a) Scan rate dependent CV of nanocomposite modified electrode, (b) cathodic current Vs square root of scan rate. Figure S4: Optimization of chronoamperometry potential of nanocomposite with 0.1 μM of glucose added in 0.1 M NaOH.

## References

[1] (2021). IDF Atlas 10th Edition. https://diabetesatlas.org/resources/?gclid=Cj0KCQjw-daUBhCIARIsALbkjSbhgiKb1E9ANA2i7J2nW3NG_kp_kAR3nQfJ3_r_NKDIBhhj7ltvcvAaAjG2EALw_wcB.

[2] Baingane A., Narayanan J.S., Slaughter G. (2018). Sensitive electrochemical detection of glucose via a hybrid self-powered biosensing system. Sens. Bio-Sens. Res..

[3] Cannon A., Handelsman Y., Heile M., Shannon M. (2018). Burden of Illness in Type 2 Diabetes Mellitus. J. Manag. Care Spéc. Pharm..

[4] Teymourian H., Barfidokht A., Wang J. (2020). Electrochemical glucose sensors in diabetes management: An updated review (2010–2020). Chem. Soc. Rev..

[5] Karyakin A.A. (2021). Glucose biosensors for clinical and personal use. Electrochem. Commun..

[6] Chaiyo S., Mehmeti E., Siangproh W., Hoang T.L., Nguyen H.P., Chailapakul O., Kalcher K. (2018). Non-enzymatic electrochemical detection of glucose with a disposable paper-based sensor using a cobalt phthalocyanine–ionic liquid–graphene composite. Biosens. Bioelectron..

[7] Wei M., Qiao Y., Zhao H., Liang J., Li T., Luo Y., Lu S., Shi X., Lu W., Sun X. (2020). Electrochemical non-enzymatic glucose sensors: Recent progress and perspectives. Chem. Commun..

[8] Tee S.Y., Teng C.P., Ye E. (2017). Metal Nanostructures for Non-enzymaticGlucose Sensing. Mater. Sci. Eng. C.

[9] Yoon J., Shin M., Lim J., Lee J.-Y., Choi J.-W. (2020). Recent Advances in MXene Nanocomposite-Based Biosensors. Biosensors.

[10] Huang K., Li Z., Lin J., Han G., Huang P. (2018). Two-dimensional transition metal carbides and nitrides (MXenes) for biomedical applications. Chem. Soc. Rev..

[11] Gogotsi Y., Huang Q. (2021). MXenes: Two-Dimensional Building Blocks for Future Materials and Devices. ACS Nano.

[12] Li M., Fang L., Zhou H., Wu F., Lu Y., Luo H., Zhang Y., Hu B. (2019). Three-dimensional porous MXene/NiCo-LDH composite for high performance non-enzymatic glucose sensor. Appl. Surf. Sci..

[13] Yang Y., Umrao S., Lai S., Lee S. (2017). Large-area highly conductive transparent two-dimensional Ti_2_CT x film. J. Phys. Chem. Lett..

[14] Ahmed B., Anjum D.H., Hedhili M.N., Gogotsi Y., Alshareef H.N. (2016). H_2_O_2_ assisted room temperature oxidation of Ti_2_C MXene for Li-ion battery anodes. Nanoscale.

[15] Xu J., Qiao X., Arsalan M., Cheng N., Cao W., Yue T., Sheng Q., Zheng J. (2018). Preparation of one dimensional silver nanowire/nickel-cobalt layered double hydroxide and its electrocatalysis of glucose. J. Electroanal. Chem..

[16] Naguib M., Mashtalir O., Carle J., Presser V., Lu J., Hultman L., Gogotsi Y., Barsoum M.W. (2012). Two-Dimensional Transition Metal Carbides. ACS Nano.

[17] Rakhi R.B., Ahmed B., Hedhili M.N., Anjum D.H., Alshareef H.N. (2015). Effect of Postetch Annealing Gas Composition on the Structural and Electrochemical Properties of Ti_2_CT*_x_* MXene Electrodes for Supercapacitor Applications. Chem. Mater..

[18] Naguib M., Mashtalir O., Lukatskaya M.R., Dyatkin B., Zhang C., Presser V., Gogotsi Y., Barsoum M.W. (2014). One-step synthesis of nanocrystalline transition metal oxides on thin sheets of disordered graphitic carbon by oxidation of MXenes. Chem. Commun..

[19] Cai K.J., Zheng Y., Shen P., Chen S.Y. (2014). TiCx–Ti_2_C nanocrystals and epitaxial graphene-based lamellae by pulsed laser ablation of bulk TiC in vacuum. CrystEngComm.

[20] Al-Mokaram A.M.A.A.A., Yahya R., Abdi M.M., Mahmud H.N.M.E. (2017). The Development of Non-Enzymatic Glucose Biosensors Based on Electrochemically Prepared Polypyrrole–Chitosan–Titanium Dioxide Nanocomposite Films. Nanomaterials.

[21] Tang W., Li L., Zeng X. (2015). A glucose biosensor based on the synergistic action of nanometer-sized TiO_2_ and polyaniline. Talanta.

[22] Pang X., He D., Luo S., Cai Q. (2009). An amperometric glucose biosensor fabricated with Pt nanoparticle-decorated carbon nanotubes/TiO2 nanotube arrays composite. Sensors Actuators B Chem..

[23] Lopes J.H., Colson F.-X., Barralet J.E., Merle G. (2017). Electrically wired enzyme/TiO_2_ composite for glucose detection. Mater. Sci. Eng. C.

[24] Dung N.Q., Patil D., Duong T.T., Jung H., Kim D., Yoon S.-G. (2012). An amperometric glucose biosensor based on a GO_x_-entrapped TiO_2_–SWCNT composite. Sens. Actuators B.

[25] Long M., Tan L., Liu H., He Z., Tang A. (2014). Novel helical TiO_2_ nanotube arrays modified by Cu_2_O for enzyme-free glucose oxidation. Biosens. Bioelectron..

[26] Feng C., Xu G., Liu H., Lv J., Zheng Z., Wu Y. (2014). Glucose biosensors based on Ag nanoparticles modified TiO_2_ nanotube arrays. J. Solid State Electrochem..

[27] Feng C., Xu G., Liu H., Lv J., Zheng Z., Wu Y. (2014). Facile Fabrication of Pt/Graphene/TiO_2_ NTAs Based Enzyme Sensor for Glucose Detection. J. Electrochem. Soc..

[28] Zhang Z., Xie Y., Liu Z., Rong F., Wang Y., Fu D. (2011). Covalently immobilized biosensor based on gold nanoparticles modified TiO_2_ nanotube arrays. J. Electroanal. Chem..

[29] Jang H.D., Kim S.K., Chang H.K., Roh M., Choi J.W., Huang J. (2012). A glucose biosensor based on TiO_2_–graphene composite. Biosens. Bioelectron..

[30] Si P., Ding S., Yuan J., Lou X.W., Kim D.H. (2011). Hierarchically structured one-dimensional TiO_2_ for protein immobilization, direct electrochemistry, and mediator-free glucose sensing. ACS Nano.

[31] Guo Q., Liu L., Zhang M., Hou H., Song Y., Wang H., Zhong B., Wang L. (2017). Hierarchically mesostructured porous TiO_2_ hollow nanofibers for high performance glucose biosensing. Biosens. Bioelectron..

[32] Muthuchamy N., Gopalan A., Lee K.-P. (2018). Highly selective non-enzymatic electrochemical sensor based on a titanium dioxide nanowire–poly(3-aminophenyl boronic acid)–gold nanoparticle ternary nanocomposite. RSC Adv..

