# Supplementary material for: Ti2C-TiO2 MXene Nanocomposite-Based High-Efficiency Non-Enzymatic Glucose Sensing Platform for Diabetes Monitoring
Source: Sensors (Basel). 2022 Jul 26;22(15):5589. doi: 10.3390/s22155589 (PMC9371085; doi:10.3390/s22155589)
Supplement: Supplementary file 1 [file sensors-22-05589-s001.zip › sensors-1776378-supplementary.pdf]

Supplementary information!

Ti_2C-TiO_2 MXene nanocomposite based high efficient non-enzymatic Glucose sensing platform for diabetes monitoring

Vinod Kumar[1†*], Sudheesh K. Shukla[2], Meenakshi Choudhary[3†], Jalaj Gupta[1],Priyanka Chaudhary[4], Saurabh Srivastava[5], Mukesh Kumar[6], Manoj Kumar[7], Devojit Kumar Sarma[7], Bal Chandra Yadav[4], Vinod Verma[1*]

[1]Stem Cell Research Centre, Department of Hematology, Sanjay Gandhi Post Graduate Institute of Medical Sciences, Lucknow-226014, UP, India

[2]Department of Biomedical Engineering, Shobhit Institute of Engineering & Technology (Deemed to-be-University), Meerut-250110, U.P., India

[3]Department of Solar Energy and Environmental Physics, The Swiss Institute of Dryland, Environmental and Energy Research, The Jacob Blaustein Institutes for Desert Research, Ben-Gurion University of the Negev, Midreshet Ben-Gurion-8499000, Israel

[4]School of Physical & Decision Sciences, Babasaheb Bhimrao Ambedkar University, (A central University) Lucknow-226025, U.P., India

[5]Department of Applied Sciences & Humanities, Rajkiya Engineering College Ambedkar Nagar (affiliated to Dr. A.P.J. Abdul Kalam Technical University, Uttar Pradesh), Ambedkar Nagar-224122, U.P., India

[6] Department of Zoology, Babasaheb Bhimrao Ambedkar University, (A central University) Lucknow-226025, U.P., India

[7]ICMR- National Institute for Research in Environmental Health, Bhopal Bypass Road, Bhouri, Bhopal - 462030, Madhya Pradesh, India

† Equal contributors

**Correspondence*:

1. vverma29@gmail.com

2. mail2vinod2@gmail.com

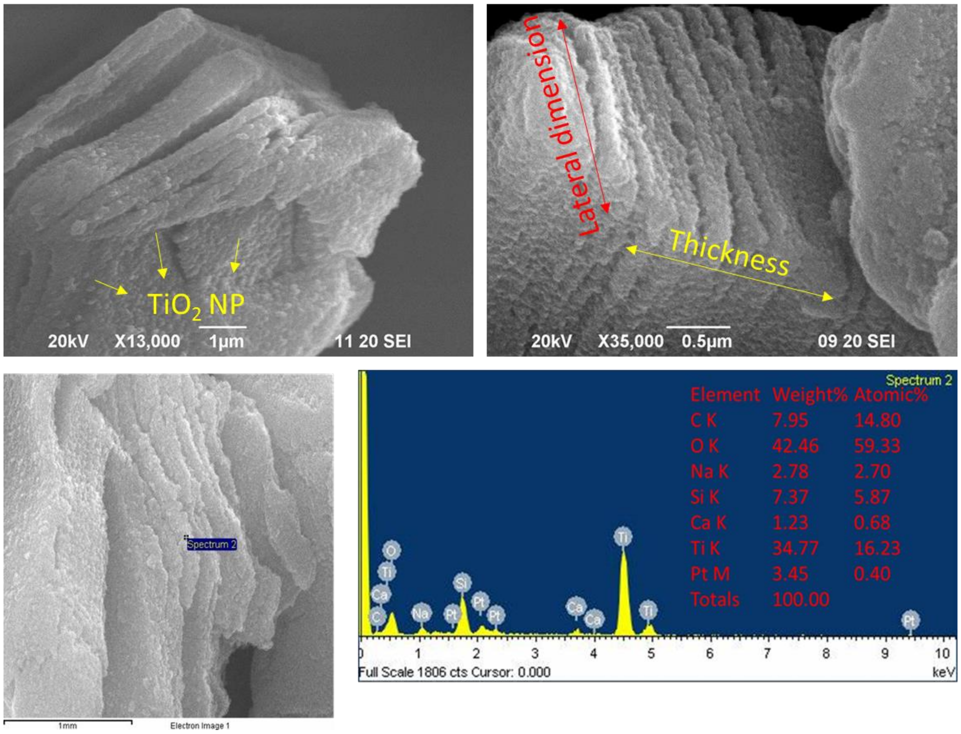

Figure S1. SEM of synthesized MXene nanocomposite and elemental analysis

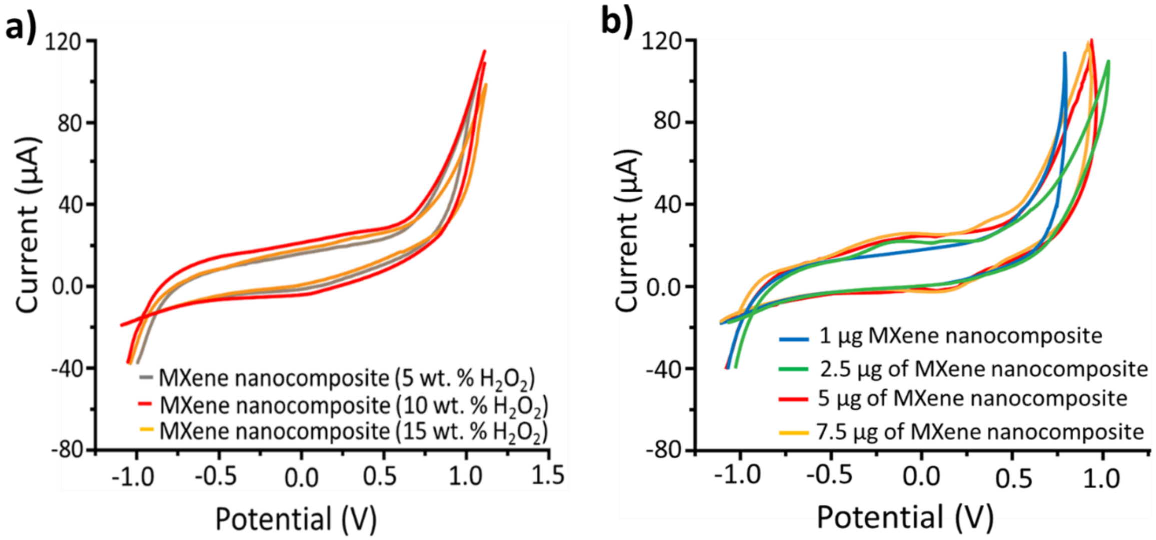

Figure S2. a) Electrochemical characterization of synthesized nanocomposites, b) Optimization of electrochemical response ofMXene nanocomposite

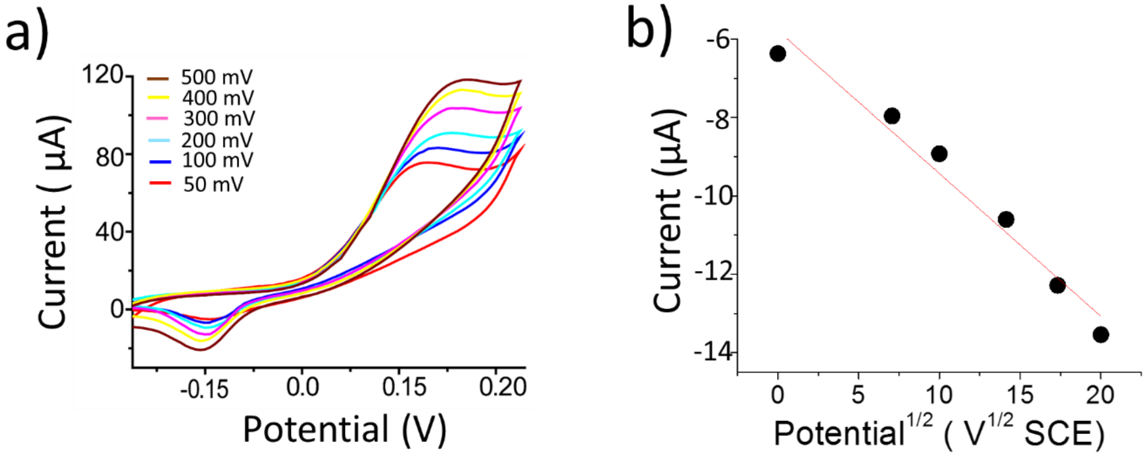

Figure S3. a) Scan rate dependent CV of nanocomposite modified electrode, b) cathodic current *Vs* square root of scan rate

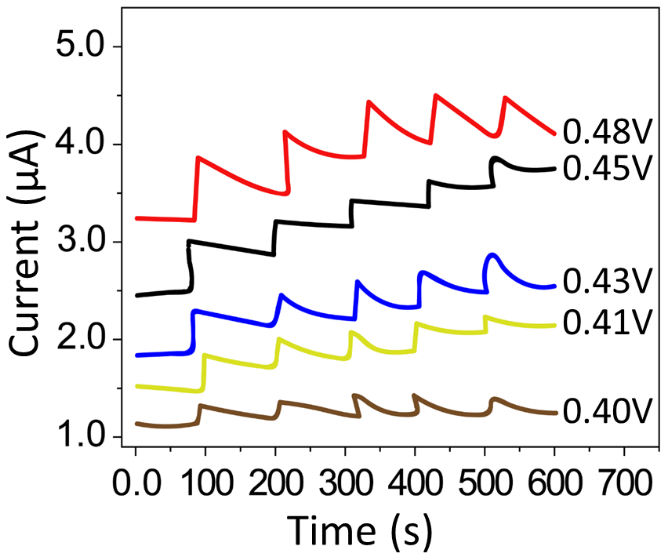

Figure S4. Optimization of chronoamperometry potential of nanocomposite with 0.1 μM of glucose added in 0.1 M NaOH
